# Surface-Modified Biochar with Polydentate Binding Sites for the Removal of Cadmium

**DOI:** 10.3390/ijms20071775

**Published:** 2019-04-10

**Authors:** Rongqi Chen, Xi Zhao, Juan Jiao, Yan Li, Min Wei

**Affiliations:** 1 College of Horticultural Science and Engineering, Shandong Agricultural University, Tai’an 271018, Shandong, China; echo_chenrq@126.com (R.C.); jjsjz@163.com (J.J.); edmonlee@163.com (Y.L.); 2 Ji’nan Academy of Agricultural Sciences, Ji’nan 250316, Shandong, China; zhaoxixinxiang@163.com; 3 State Key Laboratory of Crop Biology (Shandong Collaborative), Tai’an 271018, Shandong, China; 4 Innovation Center of Fruit & Vegetable Quality and Efficient Production, Tai’an 271018, Shandong, China; 5 Scientific Observing and Experimental Station of Environment Controlled Agricultural Engineering in Huang-Huai-Hai Region, Ministry of Agriculture, Tai’an 271018, Shandong, China

**Keywords:** biochar, modification, cadmium, cystamine, multisite

## Abstract

In this study, a surface chemical-modified rice husk biochar with abundant amino groups and disulfide bonds for the removal of cadmium was prepared using cystamine dihydrochloride as a modification ligand and glutaraldehyde as a crosslinker. The biochars were characterized by Fourier transform infrared spectrometry (FTIR), elemental analysis, scanning electron microscopy (SEM), X-ray photoelectron spectroscopy (XPS), thermogravimetry analysis (TGA), and nitrogen sorption (BET) before and after modification. The adsorption properties of the modified biochars for Cd (II) were investigated in detail via adsorption isotherm models, adsorption kinetics models, and selective adsorption experiments. The surfaces of the cystamine-modified biochars with granular nanopolymers of sufficient functional groups of primary amine and disulfide linkage rendered the biochar surface more conducive to electrostatic attraction and surface complexation. The theoretical maximum adsorption capacity of the modified biochars (81.02 mg g^−1^) was almost 10-fold greater than that of the raw biochars (8.347 mg g^−1^) for Cd (II). Besides, the cystamine-modified biochars had a better affinity for Cd (II) compared to other heavy metals (Zn, As, Cd, Co, Ni, Cr), showing six-fold greater affinity for Cd (II) than Zn^2+^. The results of this study indicate that the modification of biochars derived from rice husks shows great potential in the removal of Cd (II) from contaminated water.

## 1. Introduction

Environmental pollution and food safety are two of the most important issues of our time. Soil and water pollution, in particular, have historically impacted food safety and therefore represent an important threat to human health [1]. Among all types of pollutants reported, heavy metals are considered to present the greatest risk to food safety in China [2]. The accumulation of heavy metals is rapidly increasing, especially in farmlands with intensive agriculture and large irrigation systems [3]. According to a recent official nationwide survey in China, 39 of 55 sewage irrigation areas were contaminated by cadmium (Cd), arsenic (As), and polycyclic aromatic hydrocarbons [4]. Cadmium is not an essential nutrient element for the human body, but is an environmental pollutant that is mainly generated from industrial effluent and sludge, such as that associated with zinc mining, sewage treatment, and electroplating. Cadmium is also a teratogen and carcinogen, as well as having the potential to cause acute and chronic poisoning when ingested in excessive amounts [5,6]. The World Health Organization (WHO) has listed Cd as an important food contaminant for research, the International Agency for Research on Cancer (IARC) has classified Cd as a human carcinogen that causes serious health damage to humans, and the American Toxicology and Disease Registry (ATSDR) has listed Cd as the substance that is seventh most harmful to human health. Therefore, it is important to develop methods for the removal of cadmium from the environment.

Several methods have been proposed to remove heavy metal ions from water and soil [7,8,9,10], including phytoremediation [11], physical remediation [12], chemical remediation [13], and bioremediation [14]. In recent years, the utilization of biomass residues from agriculture as heavy metals remediation materials has received increased attention worldwide because of their environmentally friendly nature, low cost, and wide availability [15]. Biochar is produced from the incomplete pyrolysis of biomass residues such as rice husk and straw under the partial or total absence of oxygen [16,17]. Lu et al. have investigated the effects of bamboo and rice straw biochar on the mobility and redistribution of heavy metals (Cd, Cu, Pb, and Zn) in contaminated soil [18]. Moreover, Alam et al. have studied the effects of biochar and rice husk on adsorption and desorption of cadmium into soils under different water conditions [19]. However, biochar is a broad-spectrum adsorbent that lacks specificity, resulting in a low adsorption efficiency for heavy metals and a loss of essential elements for plant growth [20]. Some elegant work about biochar amendment immobilization of heavy metal in soil and reductions in its uptake and translocation to plants has been proposed [21,22,23,24,25]: These effects generally result from biochar containing not only oxygenated functional groups, but also increasing soil pH and Si concentrations in the soil solution [26,27,28,29], which has a high adsorption capacity for heavy metals [30,31,32,33,34,35]. However, this effect has been poorly investigated in contaminated water.

Modification of the surface chemistry of biochar is considered a promising and attractive way to impart biochar with specificity. Approaches to modifying biochar, including chemical modification (e.g., acid/base treatment, functional groups, surfactant modification), impregnation of mineral sorbents, steam activation, and magnetic modification, have been widely studied [36,37,38]. Chemical modification of biochar may enhance its contaminant sorption ability by creating additional and abundant sorption sites on increased surface areas, rendering biochar surfaces more conducive to electrostatic attraction, surface complexation, and/or surface precipitation, as well as enabling greater sorption affinity through stronger interactions with specific surface functional groups [39]. Since surface complexation between metals and functional groups such as carboxylic, amino, thiol, and hydroxyl groups plays an important role in metal sorption, various exogenous functional groups have been added to biochar. For example, Ma et al. have prepared poly(ethylenimine)-modified biochars as adsorbents for hexavalent chromium removal from aqueous solution aminos [40]. Chemical oxidation using HNO_3_, KMnO_4_, H_2_O_2_, H_3_PO_4_, or HNO_3_/H_2_SO_4_ mixtures has introduced acidic functional groups such as carboxylic, carbonyl, lactonic, and phenolic groups on the C surface at relatively low temperatures [41,42,43]. However, the surface complexation sites on these modified biochars were relatively single and were often only carboxyl groups or amino groups. The few studies that have investigated polydentate multisite modification of biochars had one postmodification.

**Figure 1:**
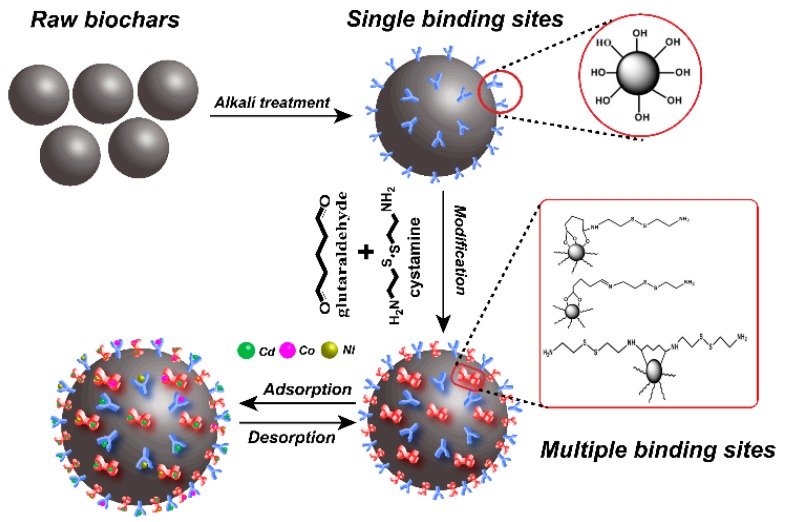
Schematic illustration of modification process of cystamine-modified biochars.

To address these problems, we used cystamine as a ligand and glutaraldehyde as a crosslinker for surface modification of alkali-treated rice husk biochar. The process for preparation of cystamine-modified biochar is shown in Figure 1. Cystamine has the chemical structure NH_2_-(CH_2_)_2_-S-S-(CH)_2_-NH_2_, and cystamine-modified biochars have abundant disulfide bonds, amino groups, and hydroxyl groups, which could provide multiple binding sites for heavy metals on the surface of raw biochars. Accordingly, cystamine-modified biochars can immobilize toxic heavy metals through the surface complexation and electrostatic interaction of heavy metals with disulfide bonds and amine groups.

## 2. Results and Discussion

### 2.1. Characterization of Raw and Modified Biochars

#### 2.1.1. FTIR of Raw and Cystamine-Modified Biochars

**Figure 2:**
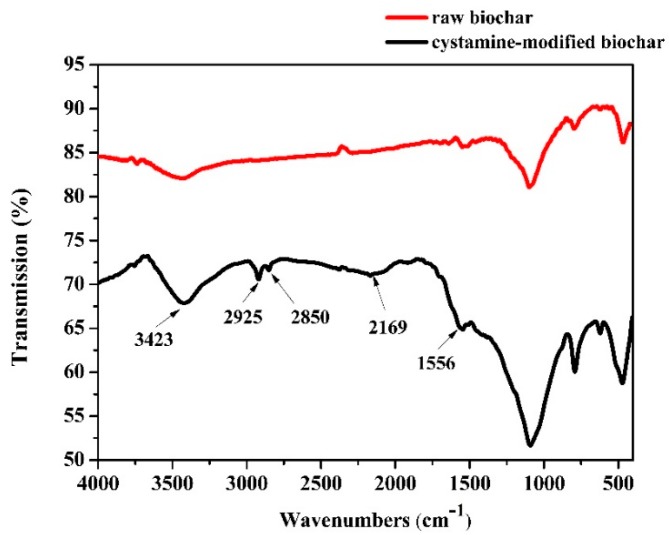
Fourier transform infrared spectroscopy (FTIR) of the raw and cystamine-modified biochars.

Changes in the functional groups of biochars before and after modification were analyzed by Fourier transform infrared spectroscopy (FTIR) (Figure 2). The peak at 3423 cm^−1^ in the spectrum of the raw biochars was assigned to an -OH stretching vibration. No obvious peaks between 3000 cm^−1^ and 2000 cm^−1^ were found in the spectra of the raw biochars. However, peaks in the modified biochar spectra were observed at 2925 and 2850 cm^−1^ and were attributed to -CH and -CH_2_ stretching, respectively. The peak at 2169 cm^−1^ was attributed to the stretching of -N=C=O bonds [44], but for the raw biochar, there were no peaks observed at 2169 cm^−1^. The modification process consisted of three steps: (1) The raw biochars were treated with NaOH solution to expose more hydroxyl groups (-OH); (2) the obtained alkaline-treated biochars were treated with glutaraldehyde solution (with the glutaraldehyde as a linking reagent), and the aldehyde groups could react with the hydroxyl groups on the surface of the alkaline-treated biochars via aldol condensation; (3) the unreacted aldehyde groups of the surface of the biochars could react with the amino groups of cystamine through a Schiff base reaction. The results of the -N=C=O bonds indicated that the cystamine was grafted onto the glutaraldehyde-modified biochar through a reaction between the aldehyde and amino groups.

#### 2.1.2. TGA of Cystamine-Modified Biochars

**Figure 3:**
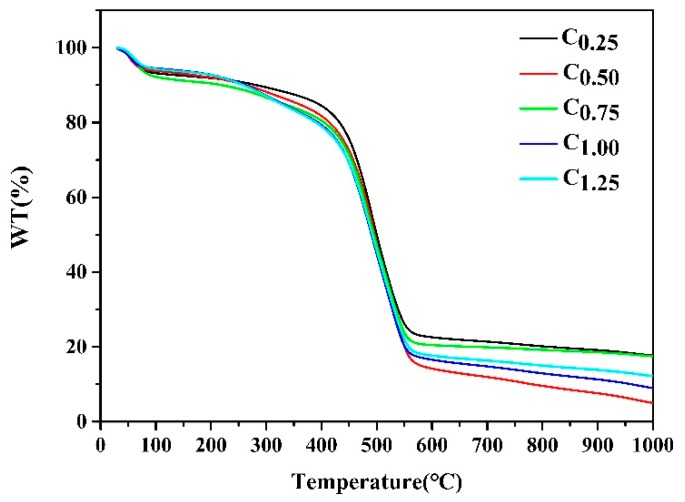
TGA analysis of different types of the cystamine-modified biochars.

The results in Figure 3 show the TGA curves of different types of cystamine-modified biochars from an ambient temperature to 1000 °C at a heating rate of 10 °C min^−1^ under a nitrogen atmosphere. The first weight loss occurred at 100 °C, which was attributed to the removal of adsorbed water, while a significant weight loss occurred at 450 °C, which could have been due to a breakdown of the polymers in the macropores of the cystamine-modified biochars.

#### 2.1.3. X-ray Photoelectron Spectroscopy (XPS) and Elemental Analysis of Raw and Cystamine-Modified Biochars

**Figure 4:**
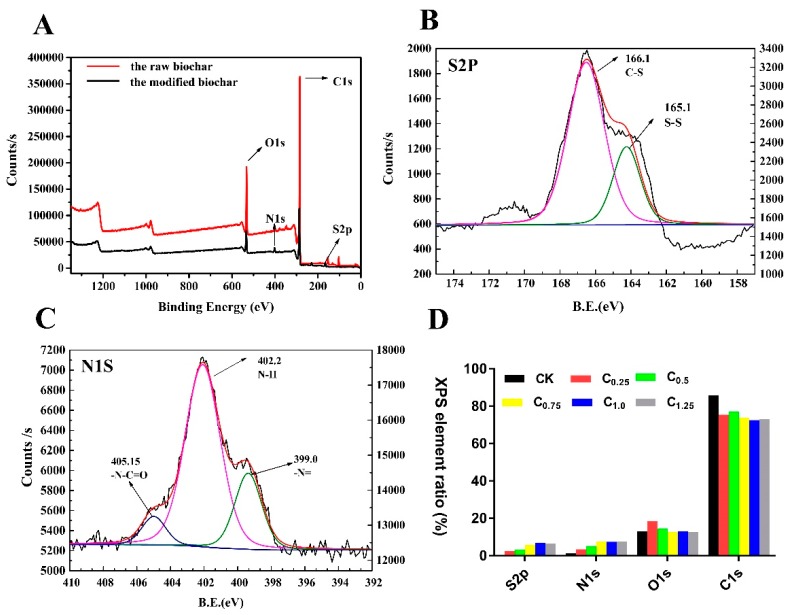
X-ray photoelectron spectroscopy (XPS) of raw and cystamine-modified biochars: (**A**) total, (**B**) S2P, (**C**) N1S, (**D**) surface element ratio.

The surface elemental compositions of the raw and modified biochars were measured by XPS. The raw biochars had almost no elemental N and S on their surfaces: However, significant increases in N and S elements were observed after modification in response to the introduction of cystamine (Figure 4D and Table S1). For further verification, the total elemental contents of the raw and modified biochars were analyzed by elemental analyzer. The total content of N and S elements in the modified biochar was obviously increased (Table 1).

Additionally, the high-resolution S2p spectrum in Figure 4B consisted of two peaks, indicating that there were two S-containing functional groups, C-S (166.1 eV) and S-S (165.1 eV). Figure 4C presents the Nls spectra of the cystamine biochars. The peak binding energy of 402.2 eV in the spectrum of the cystamine-modified biochars was attributed to amine groups, while that at 405.2 eV was assigned to the N in the amide groups (N-C=O).

Taken together, these findings indicate that aldehyde groups of glutaraldehyde on the surface of the pretreated biochars reacted with the amine groups of cystamine, which served as a polydentate ligand. The glutaraldehyde served as a bridge to graft the cystamine onto the surface of the biochars.

#### 2.1.4. SEM Image of Raw and Cystamine-Modified Biochars

**Figure 5:**
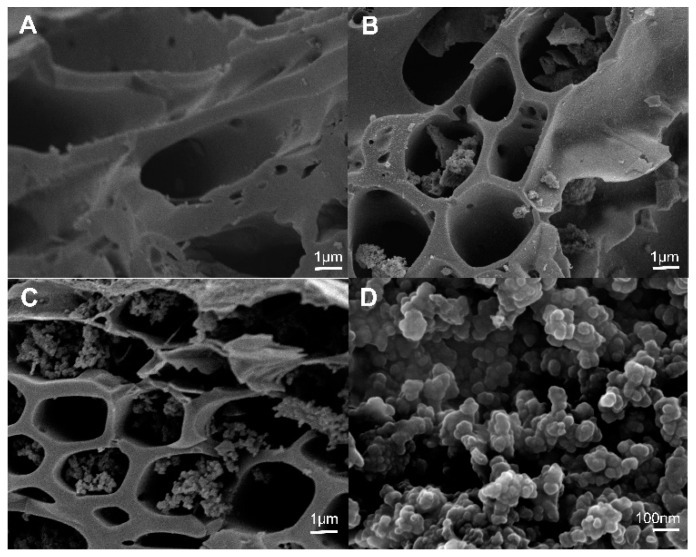
Scanning electron microscopy (SEM) of modified biochars: (**A**) Raw rice husk biochars; (**B**) raw biochar modified by the addition of 0.5 g cystamine dihydrochloride and 25 mL glutaraldehyde (2%); (**C**) raw biochar modified by the addition of 0.75 g cystamine dihydrochloride and 25 mL glutaraldehyde (2%); (**D**) modified polymer on the surface of biochars.

The surface morphology of the raw biochars was smooth (Figure 5A), and there were no polymers found in the macropores of the raw biochars (1–10 μm). However, the surface of the cystamine-modified biochars was rough, and it contained some granular polymers in the macropores (Figure 5B). As the amount of cystamine added increased, the amounts of polymers in the macropores increased (Figure 5C). As shown in Figure 5D, the polymers in the macropores of the cystamine-modified biochars were tightly bound to the surface of the macropores. Overall, the results shown in Figure 5 indicate that the surfaces of the biochars were successfully modified with cystamine via the crosslinking of glutaraldehyde.

### 2.2. Effects of pH and Ionic Strength

**Figure 6:**
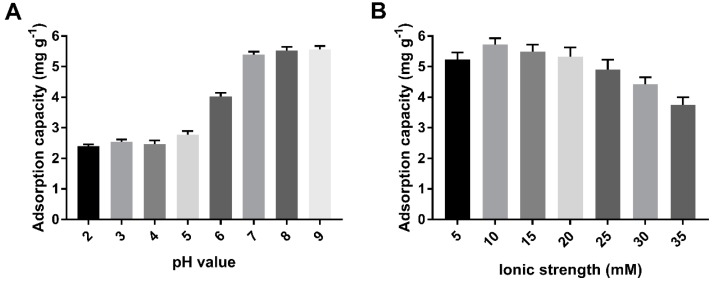
Optimization of adsorption conditions of pH (**A**) and ionic strength (**B**).

This study was carried out at a pH ranging from 2 to 9, because the formation of Cd(OH)_2_ precipitation noticeably starts at a pH greater than 9.5. The effect of the pH of Cd (II) on biochars could be explained by the electrostatic interaction between the surface of biochar and metal ions. The negatively charged surface of the biochar favored sorption toward Cd (II) with a positive charge as the pH increased from 5 to 7 (Figure 6A). A high level of Cd (II) adsorbed was maintained at pH > 7. However, at low-solution pH, the surfaces of the cystamine-modified biochars might have been positively or neutrally charged, which hindered the adsorption of positively charged metal ions.

The results shown in Figure 6B indicate that the effect of ionic strength on the adsorption capacity of cystamine-modified biochars for Cd (II) was smaller than the effect of pH values was. This may have occurred because ionic strength has a lesser effect on complexation. The adsorption capacity of biochars on Cd (II) first increased, then decreased as the ionic strength increased. This may have occurred because the increase in ionic strength enhanced the electrostatic interaction between the amino groups of the modified biochars and heavy metals at low ionic strength, while excessive ionic strength caused electrostatic repulsion, which could have reduced the electrostatic interaction between amino groups and heavy metals. As a result, we selected pH 7.0 and 10 mM NaNO_3_ solution as the optimal conditions.

### 2.3. Adsorption Isotherms of Cd (II)

**Figure 7:**
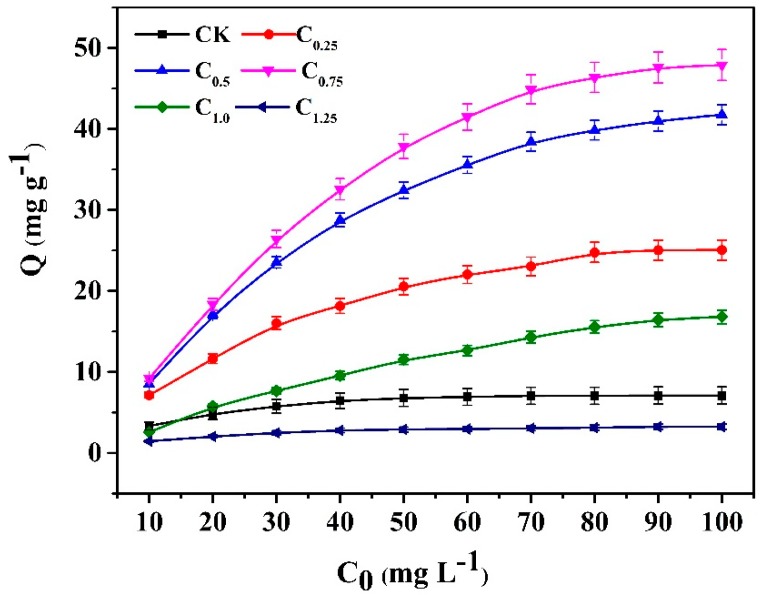
Adsorption isotherm curves of the raw and cystamine-modified biochars for Cd (II).

The isotherm adsorption curves (Figure 7) indicate that the adsorption capacity increased as the initial concentration of Cd (II) solution increased. The biochar of C_0.75_, which was modified by the addition of 0.75 g cystamine, showed the best adsorption capacity, and this was higher than that of the raw biochars over the entire range of tested concentrations. Moreover, its maximum adsorption capacity (47.87 mg g^−1^) was almost seven-fold greater (7.07 mg g^−1^) than that of raw biochars.

For the modified biochars of C_1.25_, the excess addition of cystamine resulted in the blockage of pores in biochar cavities (Table 2 and Figure S1), leading to a lower adsorption capacity than the raw biochars. For the modified biochars of C_1.0_, which had a lower adsorption capacity than the raw biochars at low concentrations of Cd (10 mg L^−1^), this may have occurred due to a reduction in biochar pore size, which was caused by modification. Conversely, the adsorption capacity of C_1.0_ increased faster than that of the raw biochars at high concentrations of Cd (20–100 mg L^−1^), which may indicate that the introduction of cystamine ligands increased selectivity for cadmium, which overcame the decrease in biochar pore size caused by the modification. The binding mechanism of modified biochars could be attributed to the primary amine groups and the disulfide linkage of cystamine, which could bind with heavy metal ions through complexation or electrostatic interaction on the surface of the modified biochars, while the adsorption of raw biochars could be ascribed to nonspecific binding.

### 2.4. Adsorption Isotherms Fitting Model of Cd (II)

In a further study, Langmuir and Freundlich isotherm adsorption models were applied to further investigate the interaction between modified biochars and Cd (II). The Langmuir and Freundlich isotherm adsorption models are expressed in Equations (1) and (2):

(1) $$q_{e}=k_{1}q_{0}C_{e}/(1+K_{T}C_{e}),$$

(2) $$q_{e}=k_{F}q_{0}C_{e}{}^{1/n},$$

where *q_e_* (mg g^−1^) and *C_e_* (mg L^−1^) are adsorption quality and solution concentration at equilibrium, respectively; *q*_0_ (mg g^−1^) is the maximum theory adsorption capacity of the Langmuir model; *K_t_* (L mg^−1^) and *K_F_* (mg g^−1^(L mg^−1^)^1/*n*^) are the adsorption constants of the Langmuir and Freundlich models, respectively; and *n* is the linearity index.

The obtained parameters (prams) of the fitting curves are presented in Table 3. The results indicate that the isothermal adsorption curve was more consistent with the Langmuir model because the values of *R*^2^ for the Langmuir model (0.980–0.997) were much higher than those for the Freundlich model (0.886–0.987). According to the theories of the Langmuir and Freundlich models, the adsorption interaction between Cd (II) and the surface of modified biochar might occur via monolayer adsorption rather than multiple-layer adsorption.

### 2.5. Adsorption Selectivity

**Figure 8:**
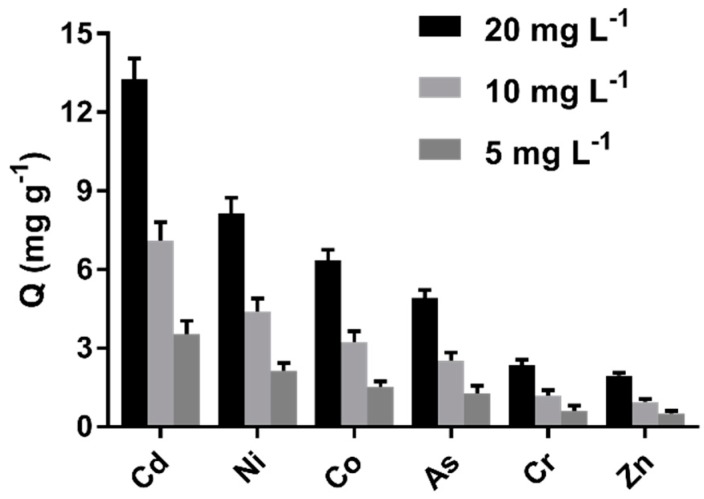
Adsorption selectivity of cystamine-modified biochars (C_0.75_) toward different heavy metal ions.

The results in Figure 8 indicate that the cystamine-modified biochars had a better affinity for Cd (II) than other heavy metals (Zn, As, Cd, Co, Ni, Cr). Indeed, the adsorption capacity of Cd (II) was six-fold greater than for Zn^2+^. Moreover, the cystamine-modified biochars had a better affinity for Co, Ni, and As, possibly because these heavy metals had sufficient empty outer orbits to bind the lone pair electrons of disulfide bonds and amine groups.

### 2.6. Adsorption Kinetics

**Figure 9:**
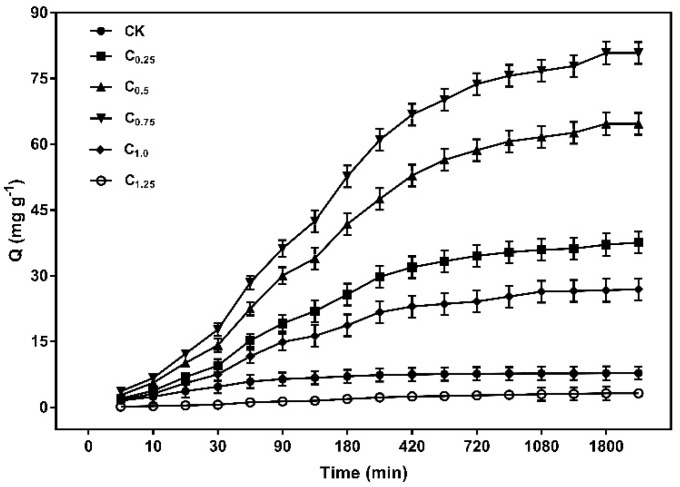
Kinetics adsorption scatterplot of the cystamine-modified biochars for Cd (II).

The results in Figure 9 indicated that the capacity of Cd (II) increased rapidly in the first 180 min, reaching more than 50% of the total adsorption. The adsorption rate then became slower, eventually reaching equilibrium at 1080 min as the adsorption time was prolonged. The higher adsorption rate at the initial stage was due to a large number of unoccupied active adsorption sites on the surface of the biochars. As the adsorption process continued, the active sites were occupied by Cd (II), leading to the adsorption rate decreasing.

### 2.7. Adsorption Kinetics Fitting Model of Cd (II)

To evaluate the mass transfer and rate-controlling process, the pseudo-first-order (PFO) and pseudo-second-order (PSO) models expressed by Equations (3) and (4) were used to analyze the kinetics data of cystamine-modified biochars:

(3) $$q_{t}=q_{e}\times (1-e^{-k_{1t}}),$$

(4) $$q_{t}=k_{2}q_{e}^{2}t/(1+k_{2}q_{e}t),$$

where *q_e_* (mg g^−1^) is the equilibrium adsorption capacity, *q_t_* (mg g^−1^) is the binding quality at different times *t* (min), and *k*_1_ (min^−1^) and *k*_2_ (g mL^−1^ min^−1^) are the rate constants of pseudo-first-order and pseudo-second-order adsorption, respectively.

Each model was evaluated according to its correlation coefficient (*R*^2^). As shown in Table 4, the *R*^2^ values of the pseudo-second-order model (0.976–0.996) were much higher than those of the pseudo-first-order (0.964–0.987) model, and the experimental value of *q_e_* was closer to the theoretical *q_e_* value obtained from the pseudo-second-order model. The dynamic model fitting results shown in Table 4 and Figure S2 indicate that the pseudo-second-order kinetics model was more suitable for the cystamine-modified biochar, and the chemical interaction between the complexation and chelation sites of the cystamine-modified biochar and Cd (II) may have been the main limiting step.

### 2.8. Repeatability Test

**Figure 10:**
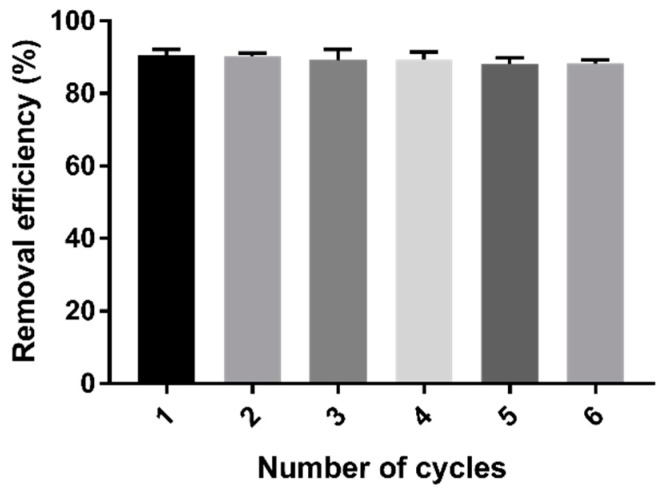
Changes in the removal efficiency of Cd (II) after six cycles of adsorption–desorption process.

The stability of the adsorption properties of cystamine-modified biochars was tested through six cycles of Cd (II) adsorption–desorption. As shown in Figure 10, repeated adsorption–desorption had little effect on the adsorption properties of the modified biochars.

### 2.9. Discussion

Furthermore, the established modification method was compared to other methods that have been reported in the literature [7,10,41,42,43]. Some features of these methods are represented in Table 5. Here, *q_e_* and *C_e_* are adsorption quality and solution concentration at equilibrium, and the enrichment factor refers to the ratio of maximum adsorption of the modified and raw biochars for Cd (II). The results indicate that cystamine-modified biochars with multiple adsorption sites had better selectivity and adsorption capacity. Further, the ideal ligand of cystamine bore an exchangeable moiety in which a disulfide linkage was assembled that could be cleaved by reduction. After the disulfide linkage was cleaved, the exposed thiol groups possessed a stronger coordination with heavy metals. Besides, the exposed thiol groups could be used for a click reaction to introduce additional functions to prepare multifunctional modified biochars [44]: This will appear in a further study.

## 3. Materials and Methods

### 3.1. Materials

Chemical reagents of an analytic grade, including cystamine dihydrochloride, glutaraldehyde, methanol, anhydrous ethanol, NaOH, HNO_3_, HCl, Cd(NO_3_)_2_, Zn(NO_3_)_2_, AS(NO_3_)_2_, Ni(NO_3_)_2_, Cr(NO_3_)_2_, and Co(NO_3_)_2_ were purchased from Sinopharm Chemical Reagent Co., Ltd. (Shanghai, China). All solutions were prepared using deionized (DI) water. The biochar, which was pyrolyzed by rice husk at 400–450 °C, was from Liao Ning Golden Future Agriculture Technology Co., Ltd. (Anshan, Liaoning Province, China).

### 3.2. The Base Treatment and Cystamine Modification Process

Rice husk biochars were ground and then passed through a 70-mesh sieve to produce granules. The raw biochars (20 g) were then dissolved in 200 mL NaOH solution (3 M), after which they were stirred for 2 h at 60 °C. The obtained alkaline-treated biochar particles were separated using a sand core funnel (G3) and washed with anhydrous ethanol and distilled water to give a neutral pH value. The alkaline-treated biochars were subsequently dried under a vacuum at 60 °C to a constant weight and then stored in a dark and dry place.

The obtained base-treated biochars (5 g) were modified as follows: Samples were dissolved in a glutaraldehyde solution (2%, 25 mL) and then stirred at 160 rpm for 4 h at room temperature. Next, different amounts of cystamine were added into the above solution (0.25, 0.50, 0.75, 1.00, 1.25 g), after which samples were stirred at 160 rpm for 12 h at room temperature. The obtained cystamine-modified biochars were then separated using a sand core funnel (G3) and washed with anhydrous ethanol and distilled water to a neutral pH value. Finally, the samples were dried under a vacuum at 60 °C to a constant weight for storage. The raw biochars and different types of the cystamine-modified biochars were referred to as CK, C_0.25_, C_0.5_, C_0.75_, C_1.0_, and C_1.25_.

### 3.3. Characterization

The rice husk biochars before and after modification were characterized by Fourier transform infrared (FTIR) spectroscopy (Nicolet, Nicolet-6700, Madison, WI, USA) at wavelengths of 400–4000 cm^−1^. The surface morphologies of the raw and cystamine-modified biochars were obtained using a Hitachi SU1510 electron microscope (SEM, Hitachi, Tokyo, Japan). The surface states of the biochars were characterized by X-ray photoelectron spectroscopy using a VG Scientific ESCALAB Mark II spectrometer equipped with two ultrahigh vacuum chambers. Thermogravimetric analysis at 30–800 °C was performed on a TGA Instrument (TGA, NETZSCH, 209F3, Selb, Germany) at a heating rate of 10 °C/min under an N_2_ atmosphere. Elemental analysis was performed on a C/H/N/S/O Elementar Analytical System (vario EL cube, Elementar, Frankfurt, Germany). The specific surface area was calculated using the Brunauer-Emmett-Teller method (Micromeritics ASAP 2405N, Norcross, GA, USA).

### 3.4. Optimization of Adsorption Conditions

#### 3.4.1. Optimization of pH

The pH of an NaNO_3_ solution was adjusted to 2–8 using HNO_3_ and NaOH at a concentration of 10 mM. Next, Cd(NO_3_)_2_ was dissolved in the NaNO_3_ solution to give a concentration of 10 mg mL^−1^, after which 0.1 g of cystamine-modified biochars were dissolved in 150 mL Cd (II) standard solution with different pH values. Samples were subsequently shaken for 8 h at 25 °C on a constant temperature water bath shaker. Next, the cystamine-modified biochars were separated by centrifugation, the supernatant was filtered with a 0.22-μm syringe filter, and the concentration of Cd (II) was determined using inductively coupled plasma mass spectrometry (ICP-MS).

#### 3.4.2. Optimization of Ionic Strength

Cd(NO_3_)_2_ was dissolved in different concentrations of NaNO_3_ solution (5–35 mM) to give a final concentration of 10 mg L^−1^, after which 0.1 g of cystamine-modified biochars were dissolved in 150 mL of Cd (II) standard solution with different pH values. Samples were subsequently shaken for 8 h at 25 °C on a constant temperature water bath shaker. Next, the cystamine-modified biochars were separated by centrifugation, the supernatant was filtered with a 0.22-μm syringe filter, and the concentration of Cd (II) was determined using inductively coupled plasma mass spectrometry (ICP-MS).

### 3.5. Equilibrium Binding Experiments

Batch adsorption experiments were conducted with 0.1 g of different types of modification biochars (CK, C_0.25_, C_0.5_, C_0.75_, C_1.0_, C_1.25_) dissolved in 100 mL of NaNO_3_ solution (10 mM) with different concentrations of Cd (II) (10–100 mg L^−1^). After shaking at 25 °C for 8 h on a constant temperature water bath shaker, the materials were separated by centrifugation, the supernatant (1 mL) was filtered with a syringe filter of 0.22 μm, and the concentration of Cd (II) was determined by ICP-MS. All experimental data were the averages of duplicate measurements, and relative errors were within 5%.

The amounts of Cd (II) ions adsorbed were presented as the adsorption capacity per unit mass (g) of the materials, and the adsorption capacity (*Q*) was calculated using the equation below:

(5) $$Q=(C_{o}-C_{t})\times \frac{V}{m}\times 10^{3}(\mathrm{mg}\;g^{-1}),$$

where *C_o_* (mg mL^−1^) is the initial concentration of heavy metal ions; *C_t_* (mg mL^−1^) is the equilibrium concentration of heavy metal ions; *V* (mL) is the volume of the heavy metal ion solution; and *m* (mg) is the mass of the cystamine-modified biochars.

### 3.6. Adsorption Selectivity toward Different Heavy Metal Ions

The capacity of cystamine-modified biochars toward different heavy metal ions was measured by incubating materials (0.1 g) in 100 mL NaNO_3_ solution (10 mM) with different concentrations of Cd (II) (5, 10, 20 mg L^−1^) at room temperature for 8 h. The materials were then centrifuged, after which the supernatant was determined by ICP-MS. Contrast adsorption experiments were also performed using Zn, Cr, As, Ni, and Co solutions under the same conditions.

### 3.7. Adsorption Kinetic Experiment

A total of 100 mL of Cd (II) solution (100 mg L^−1^) with 10 mM NaNO_3_ as the background electrolyte was prepared and transferred to a beaker flask. Next, 0.1 g of cystamine-modified biochars were added into the beaker flask. The beaker flask was then incubated at room temperature with shaking at 140 rpm. At different time intervals, the cystamine-modified biochars were centrifuged, and the suspension was filtered with a syringe filter of 0.22 μm. Finally, the concentrations of Cd (II) in the filtrate were determined by ICP-MS.

### 3.8. Repeatability Test

Adsorption–desorption experiments were conducted to assess the practical utility of the modified biochars. Briefly, cystamine-modified biochars with adsorbed Cd (II) were rinsed with 1 M HCl for 3 h, then rinsed to a neutral pH with distilled water. Next, the desorbed biochars were readsorbed on the Cd (II) solution (10 mg L^−1^, 100 mL), after which they were stirred at 160 rpm for 12 h at room temperature. The adsorption–desorption process was repeated six times, after which the removal efficiency of Cd (II) was measured using Equation (6):

(6) $$\eta (\%)=\frac{C_{o}-C_{e}}{C_{o}}\times 100,$$

where *C_o_* and *C_e_* (mg L^−1^) are the initial and equilibrium Cd (II) concentrations.

## 4. Conclusions

In this study, a novel surface modification biochar was synthesized using cystamine dihydrochloride as a modification reagent and glutaraldehyde as a crosslinker. The generated biochars had polydentate binding sites for the adsorption of Cd (II). The cystamine-modified biochars had a better affinity for Cd (II) compared to other heavy metals (Zn, As, Cd, Co, Ni, Cr), with six-fold greater affinity for Cd (II) than Zn^2+^. The theoretical maximum adsorption capacity (81.02 mg g^−1^) of the modified biochars was almost 10-fold greater than that (8.35 mg g^−1^) of the raw biochars. Moreover, the cystamine-modified biochars could maintain good adsorption properties under repeated adsorption–desorption processes. Taken together, these findings indicate that the developed method has good potential for use in the removal of Cd (II) from water.

## Figures and Tables

**Table 1:** Elemental analysis of different types of raw and cystamine-modified biochars.

Samples	Quality (mg)	N Area	C Area	H Area	S Area	N (%)	C (%)	H (%)	S (%)	C/N	C/H
CK	2.1064	390	25,811	1662	138	0.43	38.94	0.840	0.466	90.95	46.42
C_0.25_	2.0917	1071	41,020	3071	445	1.18	52.47	1.513	1.497	52.73	41.28
C_0.50_	2.1287	1424	38,837	3527	833	1.55	58.09	1.696	2.725	37.58	34.25
C_0.75_	2.0577	1597	37,403	3494	913	1.79	57.84	1.739	3.090	32.28	32.93
C_1.00_	2.0803	2047	35,253	4516	1271	2.27	53.93	2.193	4.262	23.78	24.62
C_1.25_	2.1690	2028	36,839	4779	1234	2.21	54.20	2.219	3.962	24.52	24.42

**Table 2:** The surface area, pore volume, and pore width of different types of cystamine-modified biochars.

Samples	Surface Area (m^2^/g)	Pore Volume (cm^3^/g)	Pore Width (nm)
CK	46.196	0.071	7.838
C_0.25_	41.412	0.047	5.300
C_0.50_	23.743	0.043	3.794
C_0.75_	19.065	0.041	3.390
C_1.00_	18.181	0.039	3.394
C_1.25_	16.271	0.034	1.272

**Table 3:** The parameters of the Langmuir and Freundlich adsorption models for Cd (II).

Model	Parms	Samples					
		CK	C_0.25_	C_0.50_	C_0.75_	C_1.00_	C_1.25_
Langmuir	*q*_0_	8.347 ± 0.179	35.82 ± 0.95	67.843 ± 3.82	81.02 ± 5.35	36.18 ± 2.01	3.765 ± 0.045
	*K_t_*	0.072 ± 0.07	0.026 ± 0.002	0.018 ± 0.002	0.016 ± 0.002	0.009 ± 0.008	0.063 ± 0.003
	*R* ^2^	0.980	0.995	0.990	0.987	0.997	0.995
Freundlich	*K_F_*	2.174 ± 0.348	3.031 ± 0.498	3.365 ± 0.762	3.664 ± 0.868	0.735 ± 0.113	0.851 ± 0.104
	*n*	3.667 ± 0.532	2.111 ± 0.175	1.778 ± 0.170	1.735 ± 0.169	1.447 ± 0.076	3.339 ± 0.333
	*R* ^2^	0.886	0.964	0.957	0.956	0.987	0.943

**Table 4:** Estimated kinetic model constants for Cd (II) adsorption. PFO: Pseudo-first-order; PSO: Pseudo-second-order.

Model	Param	Samples					
		CK	C_0.25_	C_0.5_	C_0.75_	C_1.0_	C_1.25_
PFO	*q_e_*	7.6 ± 0.07	35.08 ± 0.59	60.3 ± 1.0	75.28 ± 1.16	25.1 ± 0.6	2.863 ± 0.072
	*k*_1_	0.021 ± 0.001	0.008 ± 0.001	0.007 ± 0.001	0.007	0.009 ± 0.001	0.006 ± 0.001
	*R* ^2^	0.986	0.985	0.987	0.978	0.964	0.976
PSO	*q_e_*	7.858 ± 0.002	39.03 ± 0.01	68.31 ± 0.13	85.41 ± 0.43	27.73 ± 0.36	3.31 ± 0.05
	*k*_2_	0.006	0.0003	0.0001	0.0001	0.0004	0.0021
	*R* ^2^	0.976	0.993	0.994	0.995	0.994	0.996

**Table 5:** Comparison of the modification method obtained to the proposed methods in the literature.

Method	Biochar	*C_e_* (mg L^−1^)	*q_e_* (mg g^−1^)	Enrichment Factor
[7] KMnO_4_	Hickory wood	125	28.1	5.9
[10] Heating	Water hyacinth	200	70.3	-
[41] Alkali	Hickory wood	100	0.98	4.9
[42] MnO_2_	Swine manure	125	45.8	3.2
[43] H_3_PO_4_	Pine sawdust	5	20	4
Cystamine	Rice husk	80	47.9	7

## Supplementary Materials

Supplementary materials can be found at https://www.mdpi.com/1422-0067/20/7/1775/s1.
