# Supplementary material for: Surface-Modified Biochar with Polydentate Binding Sites for the Removal of Cadmium
Source: Int J Mol Sci. 2019 Apr 10;20(7):1775. doi: 10.3390/ijms20071775 (PMC6479704; doi:10.3390/ijms20071775)
Supplement: Supplementary file 1 [file ijms-20-01775-s001.pdf]

Electronic Supplementary Information: Surface-Modified Biochar with Polydentate Binding Sites for the Removal of Cadmium

Rongqi Chen, Xi Zhao, Juan Jiao, Yan Li and Min Wei

Table S1. X-ray photoelectron spectroscopy analysis of different types of cystamine-modified biochars.

Samples	S2p	N1s	O1s	C1s
CK	-	1.26	13.03	85.71
$C_{0.25}$	2.44	3.45	18.66	75.45
$C_{0.50}$	3.23	5.18	14.43	77.17
$C_{0.75}$	5.84	7.66	12.75	73.75
$C_{1.00}$	6.84	7.46	13.14	72.56
$C_{1.25}$	6.47	7.59	12.78	73.16

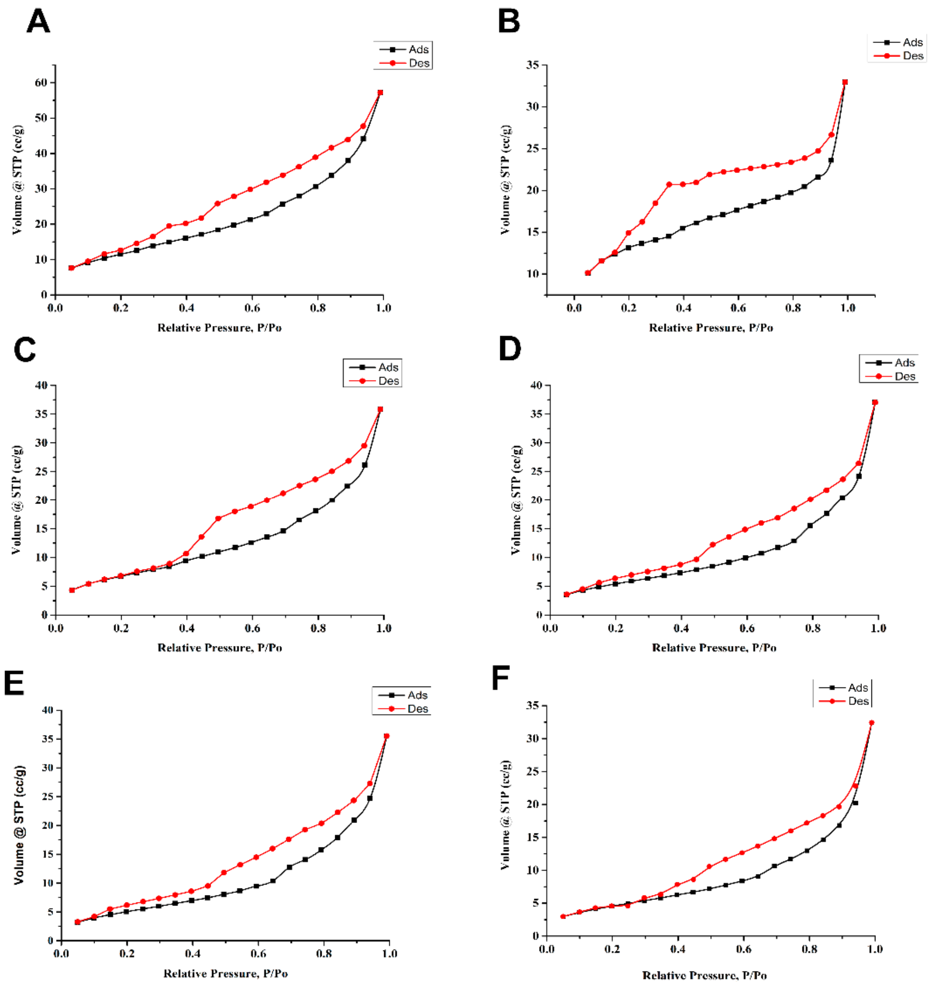

Figure S1. The nitrogen sorption (BET) curves of **(A)** CK, **(B)** $C_{0.25}$, **(C)** $C_{0.5}$, **(D)** $C_{0.75}$, **(E)** $C_{0.5}$, and **(f)** $C_{1.25}$.

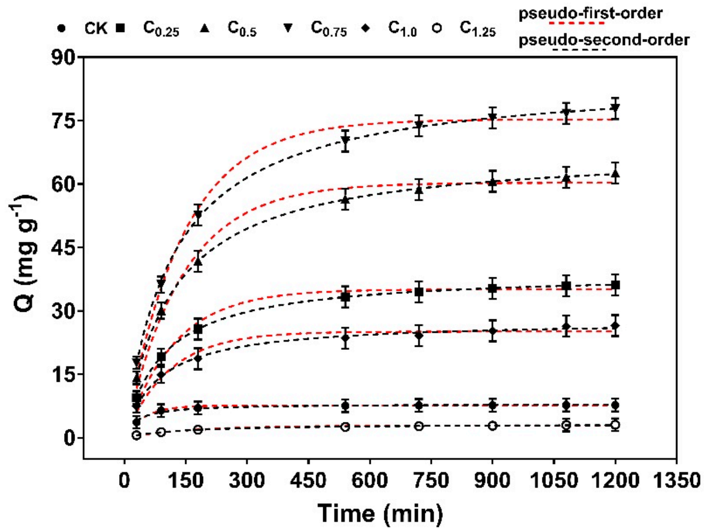

Figure S2. Kinetics adsorption fitting curves of the cystamine-modified biochars for Cd (II).
